# Cell-Based *in Vitro* Blood–Brain Barrier Model Can Rapidly Evaluate Nanoparticles’ Brain Permeability in Association with Particle Size and Surface Modification

**DOI:** 10.3390/ijms15021812

**Published:** 2014-01-24

**Authors:** Sanshiro Hanada, Kouki Fujioka, Yuriko Inoue, Fumihide Kanaya, Yoshinobu Manome, Kenji Yamamoto

**Affiliations:** 1 Research Institute, National Center for Global Health and Medicine, Tokyo 162-8655, Japan; E-Mails: fkanaya@acc.ncgm.go.jp (F.K.); backen@ri.ncgm.go.jp (K.Y.); 2 Department of Molecular Cell Biology, The Jikei University School of Medicine, Tokyo 105-8461, Japan; E-Mails: kfujioka@jikei.ac.jp (K.F.); manome@jikei.ac.jp (Y.M.); 3 Department of Anatomy, Toho University, 5-21-16 Omori-Nishi Ota-ku, Tokyo 143-8541, Japan; E-Mail: yuriko.inoue@med.toho-u.ac.jp

**Keywords:** blood-brain barrier, nanoparticles, cell-based assay, permeability coefficient

## Abstract

The possibility of nanoparticle (NP) uptake to the human central nervous system is a major concern. Recent reports showed that in animal models, nanoparticles (NPs) passed through the blood–brain barrier (BBB). For the safe use of NPs, it is imperative to evaluate the permeability of NPs through the BBB. Here we used a commercially available *in vitro* BBB model to evaluate the permeability of NPs for a rapid, easy and reproducible assay. The model is reconstructed by culturing both primary rat brain endothelial cells and pericytes to support the tight junctions of endothelial cells. We used the permeability coefficient (*P*_app_) to determine the permeability of NPs. The size dependency results, using fluorescent silica NPs (30, 100, and 400 nm), revealed that the *P*_app_ for the 30 nm NPs was higher than those of the larger silica. The surface charge dependency results using Qdots^®^ (amino-, carboxyl-, and PEGylated-Qdots), showed that more amino-Qdots passed through the model than the other Qdots. Usage of serum-containing buffer in the model resulted in an overall reduction of permeability. In conclusion, although additional developments are desired to elucidate the NPs transportation, we showed that the BBB model could be useful as a tool to test the permeability of nanoparticles.

## 1. Introduction

Nanomaterials have been a key material for electronics [1], photonics [2], and biotechnology [3–5] during the last few decades. Inorganic nanoparticles (NPs) are new materials that have great promise for practical applications in medical use and cosmetics, among other uses [6,7]. For example, silica NPs are basic materials for food additives [8], and titania (TiO_2_) is used in sun protection creams [9]. Nanoparticles also have great potential in the pharmaceutical field, as they have a special interaction with biological systems in accord with the specific size or surface forces [10,11]. Because it is not yet known how nanoparticles interact with the human body, investigations are necessary to determine these interactions to assist in future biological applications, such as drug delivery systems [12–14] and bio-imaging [15–18].

Although questions have been raised about the safety of various new technologies such as nanoparticles, there is so far no clear-cut clinical evidence for nanoparticle-related health damage. Since the engineering of nanomaterials is progressing rapidly and nanomaterials are being released on the market, the risks of nanomaterials must be examined further. Many research groups are actively investigating “nano-risks”, particularly concerning oxide nanoparticles such as silica or titania [19–22], and semiconductors, such as Qdots [23–25] or silicon [26,27]. It has been revealed that pulmonary damage by silica NPs is enhanced by nanoscale processing technologies [28].

Several research groups have investigated whether nanoparticles are transported into the central nervous system (CNS) as a new target for pharmaceuticals [29–35] or toxicological analysis [36,37]. The mechanism of nanoparticles’ transportation into the CNS is thought to be via the olfactory epithelium [38,39], or systemic blood circulation [40]. Brun *et al.* reported that TiO_2_ nanoparticles accelerated the breakdown of the tight junctions of endothelial cells and the blood–brain barrier (BBB) [36]. Yamashita *et al.* observed that 70 nm silica nanoparticles accumulated in the brain parenchyma in a fetal mouse model [41]. Our collaborative group also reported that in mouse brain, the intraperitoneal administration of Qdots was detected not only in the blood vessels, but also outside them [40]. Some groups predict nano-damage in the brain; they contend that nanoparticles accumulate in the brain and may cause cellular toxicity or brain diseases such as Alzheimer’s disease [42,43], but this has not been established.

There are two important issues to keep in mind when evaluating “nano-risks”. One is whether nanoparticles are transported into the body and accumulate in the organs, and another is whether nanoparticles are toxic [44]. It is known that small particles interact with cell layers because of their higher surface volume ratio compared to that of bulk materials, and that they can damage endothelial cells easily. However, few studies have addressed the question of whether nanoparticles are transported into the parenchyma directly through the vascular walls, especially to the brain.

An easy and quick tool for assaying nanoparticle permeability through the BBB is needed. A cell culture based model is a candidate for the quick assessment of nanoparticles’ transportation, as a biomimetic model [37]. In the present study, we newly applied an *in vitro* BBB model that is reconstructed by co-culture systems [45,46] to test the nanoparticles’ permeability into the brain. This BBB model is reconstructed by both primary endothelial cells and pericytes, which lead to much stronger tight junctions of the BBB and make reproducible evaluation of NPs permeability possible. Using the BBB model, we evaluated three different sizes of silica NPs and three differently surface-charged Qdots for their transportation through the BBB.

## 2. Results and Discussion

Nanoparticles have specific interactions with biological systems. For example, surface molecules of NPs enhance the cellular activation [47] and change the cytotoxicity [48]. The size of NPs also influences the cellular uptake or intracellular localization of NPs [49]. Some research groups reported that around 50 nm NPs can incorporate into cells more easily than other ranges in size of NPs, by receptor-mediated endocytosis [10,50]. Thus, evaluation of NPs transportation into the brain is a very important issue for, not only the toxicological aspect, but the pharmaceutical applications of NPs.

### 2.1. Size Dependency of BBB Permeability by Silica NPs

**Figure 1.**
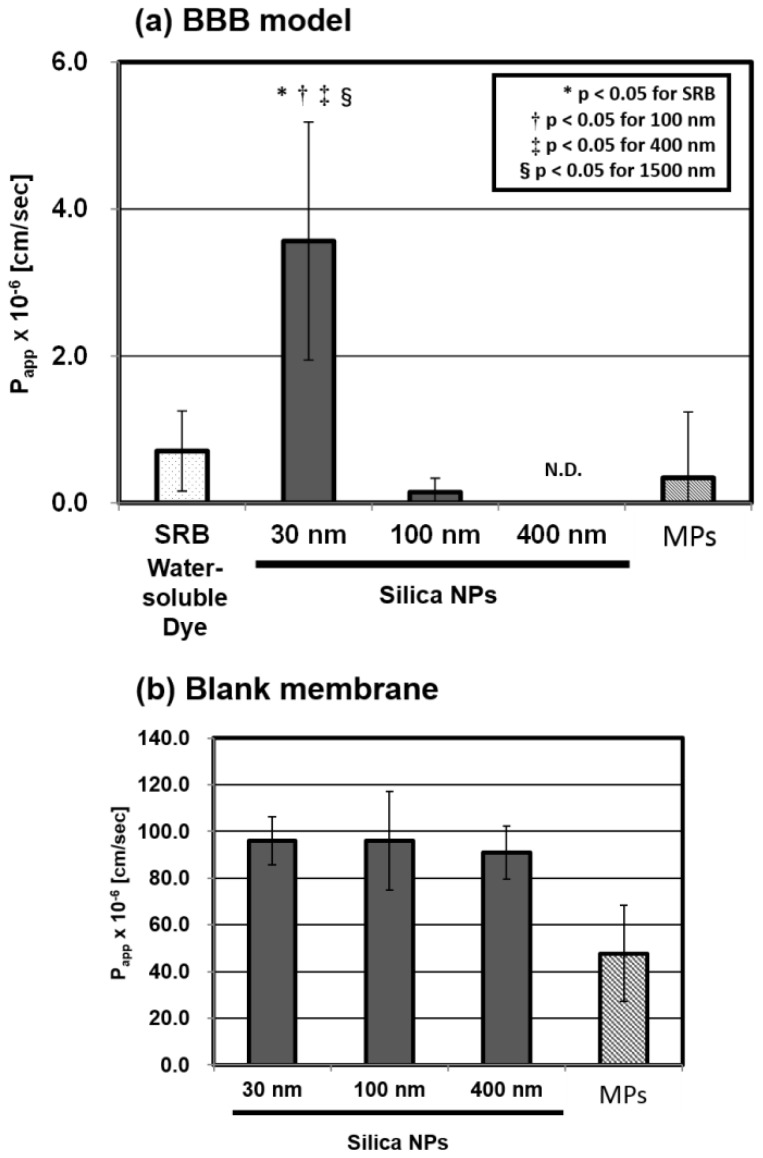
Permeability coefficients (*P*_app_) using: (**a**) *in vitro* BBB model; and (**b**) blank membrane, for the four sizes of fluorescent silica particles (30, 100, 400 nm and micro-particles (MPs)) and sulforhodamine B (SRB). We tested the silica particles at the concentration of 1.0 mg/mL and SRB at the concentration of 0.87 μM, in 30 min cultures. The *P*_app_ was calculated by the formula given in Section 3.4. These values are the means ± SD of: (**a**) 7–8 wells from three independent experiments; and (**b**) six wells from three independent experiments.

**Scheme 1.**
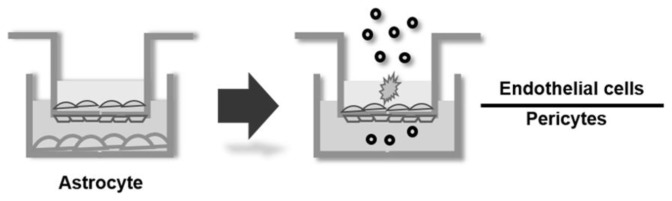
Blood–brain barrier (BBB) permeability assay using cell-based *in vitro* BBB model.

We executed the BBB permeability assay according to the procedure described in Section 3.3 (Scheme 1). After the assay, we measured the concentration of NPs and estimated the permeability coefficient (*P*_app_) calculated by the formula given in Section 3.4. We compared the permeability through the BBB model of the four different sizes of silica particles (30, 100, 400 nm, and the micro-particles (MPs)). As depicted in Figure 1a, the size-dependency results revealed that the 30 nm silica NPs were transported through the BBB model and the *P*_app_ is (3.56 ± 1.62) × 10^−6^ cm/s, which is significantly higher than those of the 100 nm or larger silica (0.14 ± 0.19, N.D., and 0.34 ± 0.90 × 10^−6^ cm/s), which hardly passed through the BBB model. The blank assay using membrane without cells showed that the *P*_app_ of three kinds of NPs was almost the same value, while the *P*_app_ of MPs was significantly lower than those of the series of NPs. This means that the membrane does not interfere with the permeability of NPs in the ranges of 30 to 400 nm.

Our results in Figure 1 indicate that: (1) 30 nm silica NPs can be transported into the brain through the BBB; (2) the *P*_app_ ((3.56 ± 1.62) × 10^−6^ cm/s) is equivalent to the value of drug molecules validated by using the same BBB model [45], which are known as CNS drugs with relatively low brain-to-plasma ratios, such as sulpiride (0.078) or midazolam (0.23) [51]; and (3) the threshold of size dependent permeability was significantly detected between 30 and 100 nm, using the BBB model. The size dependent transportation into the brain is similar to animal experiments, previously reported by other groups [41,52].

In this study, we used a commercially available *in vitro* BBB model purchased from PharmaCo-Cell Co. (Nagasaki, Japan). This model is known to be applicable to estimate a CNS drug permeability in a short time (30 min) and it is already confirmed that the estimated permeability coefficient is correlated with *in vivo* permeability [45,46]. Some research groups also applied cell-based BBB model for evaluation of NPs permeability through BBB [37,53]. However, their BBB models are constructed by only brain vascular endothelial cells [53] or cell lines [37]. Moreover, the most crucial point we considered, is that they did not directly measure nanoparticles’ permeability: the former estimated the permeability coefficient of other fluorescent molecules following exposure to Ag-NPs [53] and the latter failed to determine the permeability coefficient of 50 nm silica nanoparticle [37]. As far as we know, it is the first report that the *P*_app_ of the nanoparticles is directly estimated, comparing size dependency using *in vitro* BBB model.

### 2.2. Microscopic Analysis of the BBB Model

**Figure 2.**
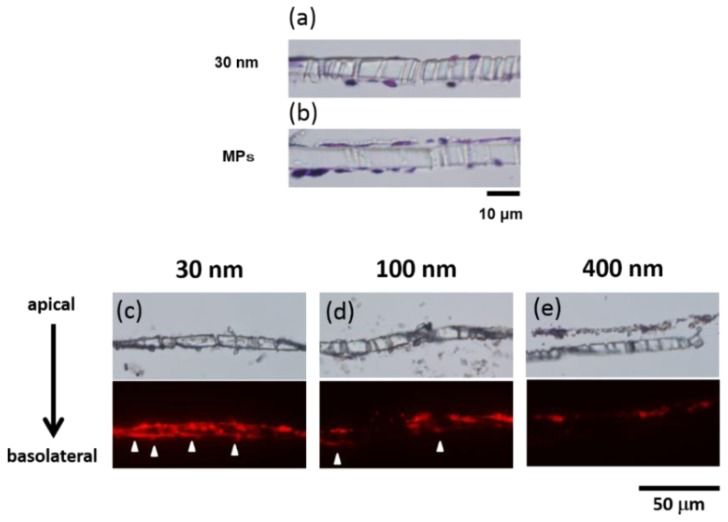
Microscopy analysis of the BBB model. In (**a**) and (**b**), we stained by H&E after supplementation of the 30 nm silica and the MP silica, respectively; in (**c**–**e**), we confirmed red fluorescence of the silica accumulation in the BBB model’s cell layers by fluorescent microscopy (30, 100, and 400 nm).

To confirm the histology of the BBB cell layers, we executed hematoxylin and eosin (H&E) staining. In the vertical sections of the cell layers (Figure 2a,b), thinning of the endothelial cell layers was observed by exposure to the 30 nm NPs, compared with the MPs, indicating “loosening” of the tight junction of the BBB cell layers.

The fluorescence of the NPs confirmed that the 30 nm silica NPs accumulated not only on the apical side but also on the basolateral side of the cell layers; the 100 nm NPs accumulated on the basolateral side slightly, and the 400 nm NPs accumulated only on the endothelium side (Figure 2c–e).

We estimated the collection ratio of the nanoparticles from the assay buffer both on the apical and basolateral sides (Table 1) as an indication of NPs uptake in the BBB cell layers. The 30 nm NPs has a lower collection ratio than the 100 and the 400 nm NPs, regardless of the adsorption of the NPs by blank membrane. In light of these results, we assume that NPs with a specific size are taken up into the BBB cell layers more and affect not only the endothelial cell, but also the pericytes, thereby enhancing the BBB permeability through the transcytotic or paracellular pathway [54].

### 2.3. Detailed Evaluation of BBB Permeability by Silica NPs

#### 2.3.1. Concentration Dependency of the *P*_app_

**Figure 3.**
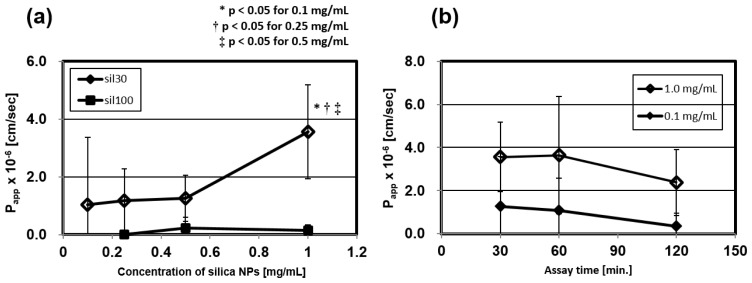
Concentration and time dependent permeability of silica nanoparticles. (**a**) We evaluated the *P*_app_ at four concentrations (0.1, 0.25, 0.5, and 1.0 mg/mL) of the 30 and 100 nm silica NPs; (**b**) We evaluated the *P*_app_ of the 30 nm silica NPs (0.1 and 1.0 mg/mL) at three time-courses (30, 60, and 120 min). These values are means ± SD of 6–12 wells from two or three independent experiments.

When we assume that NPs are used by intravenous administration as a drug carrier of molecular-targeted therapy or a diagnostic reagent, 1.0 mg/mL, adopted in the research, is an upper limit physiologically. Therefore, regarding the 30 and 100 nm silica NPs, we assayed their BBB permeability by using concentrations from 0.1 to 1.0 mg/mL. The *P*_app_ of the 30 nm silica had almost the same value from 0.1 to 0.5 mg/mL, but it was enhanced at a concentration of 1.0 mg/mL (Figure 3a), while those of the 100 nm silica were stable. These results indicate that administration of high concentrations of the 30 nm NPs enhances nanoparticles’ transportation into the brain by an additional mechanism. One of the possible reasons could be that the BBB cell layers, affected by the high concentration of the NPs, undergoes membrane damage [55], or that the high concentration gradient between blood and brain enhances the BBB permeability [54].

#### 2.3.2. Time Dependency of the *P*_app_

We evaluated the effect of assay times on the permeability through the BBB model of the 30 nm silica NPs at the concentration of 0.1 and 1.0 mg/mL. We compared the permeability at three different assay times (30, 60, and 120 min). Figure 3b showed that these *P*_app_ did not change significantly at these assay times. In light of these results, it appears that the BBB break down by long-term exposure is negligible. However, there is a possibility that long-term assay reduces the functionality of the BBB, as the *P*_app_ of the 30 nm NPs tends to slightly decrease depending on time. If NPs accumulate in the BBB for a long time, they might enhance cellular damage by biological stress such as an oxidative stress [56] and cause the BBB disruption.

### 2.4. Surface-Charge Dependency of BBB Permeability Using the Qdots

**Figure 4.**
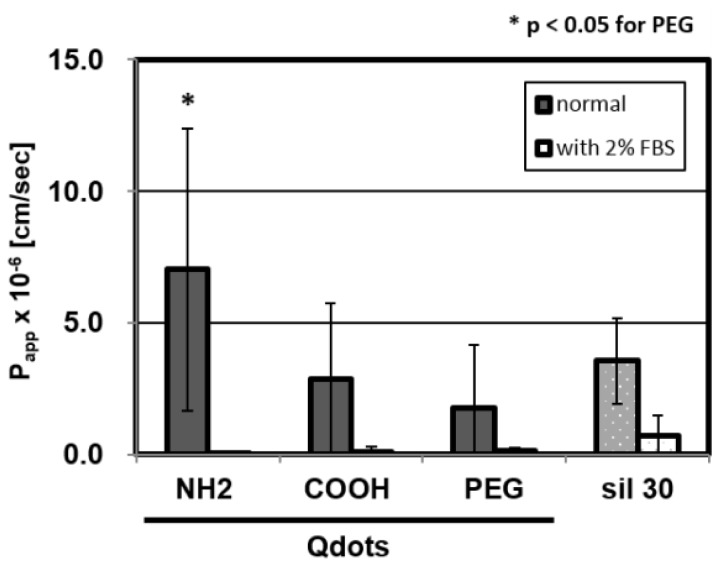
Permeability of three different electrically charged Qdots (-COOH, -PEG, -NH_2_), with or without serum. We tested these *P*_app_ at a concentration of 40 nM in 30 min cultures. These values are means ± SD of 6–8 wells from two or three independent experiments.

We evaluated the effect of surface-charge on the permeability through the BBB model using quantum dots modified with anionic, cationic and neutral functional groups, whose sizes are nearly the same as that of the 30 nm silica NPs (Table 3). Figure 4 showed that they have different permeability capacities. The cationic amino-Qdots tend to be transported onto the basolateral side through the BBB model with higher permeability than anionic carboxyl- or neutral PEGylated Qdots. We assume that, as amino NPs interact with the cell surface, which is composed of anionic phospholipids, they are transported through the BBB by the paracellular pathway, while the carboxyl NPs are known to be mainly incorporated into cells by endocytosis [57] and retained in the BBB cell layers for a while; in fact, as depicted in Table 2, the collection ratio for carboxyl-Qdots is much lower than that of the amino-Qdots.

On the other hand, in a previous *in vivo* experiment, anionic Qdots were reported to accumulate in the brain more than the other Qdots by intravenous injection with the same concentration [25]. The difference between *in vitro* and *in vivo* could be explained by the difference of the plasma half-life of each Qdots; carboxyl-Qdots is retained in blood for a longer time than PEGylated- or amino-Qdots, thus the BBB is, consequently, exposed to the cationic NPs at a higher concentration than the others.

In this study, we executed the assay using PBS-based assay buffer, according to the standard procedures. However, NPs interact with various proteins in blood. For example, a recent study reports nanoparticles are known to form “protein corona” in blood by absorbing serum proteins, which alters the cell-NPs interaction [58]. Therefore, we examined the effect of serum on BBB permeability using 2% FBS-containing buffer. Results showed that *P*_app_ greatly decreased (Figure 4) and the particle collection ratio was largely enhanced (Table 2). Many studies also reported that serum protects cell damage in long-term culture like 3–24 h culture [59,60]. This is probably because serum mitigates the interaction between cellular surface and NPs. For investigating the effect of serum protein on the NPs transportation, it is indispensable to optimize the long-term assay using the BBB model. In future, improved culture system will be desired, such as a serum-containing assay medium to maintain the functionality of the BBB cell layers, a low-concentration detection method to compare the permeability in a narrow size range of NPs, and so on.

## 3. Experimental Section

### 3.1. Nanoparticles

Three sizes of unmodified fluorescent silica NPs (Sicastar^®^-red 30, 100, 400 nm) and micro particles (Sicastar^®^-red 1500 nm) were purchased from Micromod Partikeltechnologie (Rosstock, Germany). Surface-modified cadmium selenide (CdSe)-based quantum dots (Qdot^®^ ITK™ carboxyl, Qdot^®^ ITK™ amine (PEG), and Qtracker^®^ non-targeted quantum dots) were purchased from Invitrogen (Carlsbad, CA, USA). All nanoparticles were suspended in phosphate-buffered saline (PBS)-based assay buffer, including glucose, and HEPES. We measured their primary size, z-average size, polydispersity index (PdI), and z-potential by dynamic light scattering (DLS) with a Zetasizer Nano ZS system (Malvern, Worcestershire, UK). The results of the characterization of nanoparticles are summarized in Table 3.

### 3.2. The Rat Blood–Brain Barrier (BBB) *in Vitro* Model

We used an *in vitro* rat BBB model (RBT-24H, BBB Kit™) purchased from PharmaCo-Cell Company Ltd. (Nagasaki, Japan). The BBB model is reconstructed by culturing both primary rat brain microvascular endothelial cells and rat brain pericytes separated by a macroporous Millicell^®^ membrane (24 wells, pore size: 3.0 μm, Millipore, Bedford, MA, USA), which are precultured with rat astrocytes to support the tight-junctions of the BBB cell layers before executing the BBB permeability assay.

### 3.3. Experimental Conditions for the BBB Permeability Assay

We pre-incubated the BBB models at 37 °C in 5% CO_2_ conditions for 4–5 days, establishing strongly reconstructed tight-junctions in the BBB models. We measured the trans-endothelial electrical resistance (TEER) to confirm the functionality of the tight-junctions. Our assays were carried out using the BBB cell layers with TEER values in the range of 150 to 300 W cm^2^. We added 0.9 mL PBS-based assay medium to blank 24-well culture plates and, after rinsing the BBB cell layers with the assay medium, the cell culture inserts on which the BBB models reconstructed were replaced to the plates, and then we added nanoparticles, suspended in 0.2 mL assay medium, to the apical side of the BBB layers and cultured the model for 30, 60 or 120 min. We compared the transport capacity among the 30, 100, and 400 nm silica NPs and the MPs at concentrations from 0.1 to 1.0 mg/mL. The efficacy of surface modification was compared using three electronically different charged Qdots at the concentration of 40 nM. We measured the *P*_app_ of sulforhodamine B (*M*_w_: 588.66; SRB), as a non-specific transport marker, at the concentration of 0.87 μM, which is the same range of the fluorescent intensity as 1.0 mg/mL silica NPs.

### 3.4. Calculation of Permeability Coefficient (*P*_app_)

We executed these tests over each period and at the end of the assay, we collected the medium from both the apical and the basolateral sides of the BBB model and we measured the NP concentration in the medium by determining the fluorescence intensity, based on the analytical curves of each fluorescent particles with a fluorescent microplate reader (Beckman Coulter, Indianapolis, IN, USA). To evaluate the transportation capacity, we used the apparent permeability coefficient (*P*_app_), which is calculated by following formula:

(1) $$P_{\text{app}}=\frac{V}{A\times {\left[C\right]}_{\text{apical}}}\times \frac{\Delta {\left[C\right]}_{\text{basolateral}}}{\Delta t}$$

[*C*]_apical_: initial concentration of fluorescent nanoparticles in apical side; *Δ* [*C*]_basolateral_: differential concentration of fluorescent nanoparticals in basolateral side; *A*: surface area of membrane; *V*: medium volume in basolateral side.

### 3.5. Histology and Fluorescent Microscopy Experiments

For the histological analysis, we fixed the BBB layers with the membrane in 4% PFA buffer and sliced from the paraffin mold to make vertical sections. Then, we executed Mayer’s H&E staining. For the fluorescent imaging observation of the BBB layers with the membrane, the paraffin in the slices was washed out with xylene and repeated ethanol washes. Then, we observed red fluorescence of the silica NPs on the slices by fluorescent microscopy (excitation wavelength 530–550 nm, emission wavelength 570 nm).

### 3.6. Statistical Analysis

We performed the statistical analysis using Tukey-Kramer’s test for the comparison between each experimental group. We considered *p*-values < 0.05 significant.

## 4. Conclusions

We evaluated the permeability of NPs through the blood–brain barrier using a cell-based *in vitro* BBB model. The 30 nm silica nanoparticles, especially at the high concentration, were transported thorough the BBB model, mirroring the same result reported in an animal model. The *in vitro* BBB model can be a useful tool for nano-assays as an alternative to *in vivo* models. This is the first step in evaluation of NPs permeability thorough BBB, using the cell-based *in vitro* model. In future, we have to further improve culture systems to elucidate the mechanism of the NPs transportation.

## Figures and Tables

**Table 1.** Results of particle collection ratio in the BBB assay of silica (1.0 mg/mL).

Silica		30 nm	100 nm	400 nm	MPs/1500 nm
Particle collection ratio (%)	BBB	61.0 ± 9.3	77.2 ± 6.5	68.9 ± 9.0	32.3 ± 12.3
	Blank	100 ± 1.7	98.1 ± 4.7	96.3 ± 6.5	75.8 ± 8.0

All data are the average ± SD of 6–8 wells from two or three independent experiments.

**Table 2.** Results of particle collection ratio in the BBB assay of Qdots (40 nM).

Qdot		Carboxyl	PEGylated	Amino
Particle collection ratio (%)	without serum	73.8 ± 4.3	82.0 ± 6.0	97.0 ± 3.2
	with serum	101.7 ± 0.3	98.6 ± 4.1	98.2 ± 0.1

All data are the average ± SD of 6–8 wells from two or three independent experiments.

**Table 3.** Characterization of nanoparticles.

Particle	Peak Diameter (nm)	Z-Average Diameter (nm)	PdI (−)	Zeta Potential (mV)
Silica				
30 nm	32.0 ± 1.1	29.5 ± 0.5	0.162 ± 0.013	−15.6 ± 1.2
100 nm	137.0 ± 1.5	129.7 ± 1.4	0.042 ± 0.005	−19.7 ± 1.05
400 nm	481.4 ± 22.1	460.3 ± 10.1	0.164 ± 0.030	−21.8 ± 2.4
MP/1500 nm	367.4 ± 157.1		0.869 ± 0.080	−29.3 ± 2.1
Qdots				
Carboxyl	49.85 ± 7.8		0.49 ± 0.028	−32.0 ± 1.3
PEGylated	32.9 ± 9.07		0.31 ± 0.028	−3.6 ± 2.1
Amino	47.9 ± 3.0		0.44 ± 0.005	−7.3 ± 3.8

We measured silica NPs and Qdots in PBS-based assay buffer (1.0 mg/mL or 40 nM, respectively) with dynamic light scattering. All data are the average ± SD of three independent measurements.
